# Morphology of the Temporomandibular Joints Regarding the Presence of Osteoarthritic Changes

**DOI:** 10.3390/ijerph17082923

**Published:** 2020-04-23

**Authors:** Marcin Derwich, Maria Mitus-Kenig, Elzbieta Pawlowska

**Affiliations:** 1 Department of Orthodontics, Medical University of Lodz, 90-419 Lodz, Poland; elzbieta.pawlowska@umed.lodz.pl; 2 Department of Prophylaxis and Experimental Dentistry, Jagiellonian University in Krakow, 31-008 Krakow, Poland; maria.mitus@interia.pl

**Keywords:** temporomandibular joint, osteoarthritis, osteoarthritic changes, cone-beam computed tomography

## Abstract

(1) Osteoarthritis, the most common disease of the temporomandibular joints (TMJs), is diagnosed by clinical and radiographic examination. Cone beam computed tomography (CBCT) is a method of choice for the imaging of osteoarthritic changes. The objective was to compare the morphology of the TMJs in CBCT images regarding the number of the osteoarthritic changes diagnosed in the area of the condyle. (2) A total of 105 patients participated in the study; their 210 TMJs were allocated into one of three groups regarding the number of diagnosed osteoarthritic changes: 1 (none or 1 type), 2 (2 types), 3 (3 or more types). The morphology of the TMJ was examined for each TMJ in the CBCT images. Statistical analysis was performed with STATISTICA version 12.0. The statistical significance level was *p* = 0.05 for all the measurements included. (3) The articular surface flattening was the most common type of the osteoarthritic changes (90%). The condylar A-P dimension differed significantly among the groups (*p* = 0.0001). The bigger the number of osteoarthritic changes diagnosed in one joint, the smaller the condylar A-P dimension that was observed. (4) The temporomandibular joints’ osteoarthritic changes occur very often, even among asymptomatic patients. The increased number of osteoarthritic changes seems to have an impact on the condylar anteroposterior dimension.

## 1. Introduction

Osteoarthritis is the most common disease of the temporomandibular joints (TMJs), which occurs more frequently in women [1,2,3,4]. The greater predisposition to this disease among the female sex may be affected by the estrogen receptor alpha polymorphism [5,6,7,8]. It is estimated that among patients with the temporomandibular joint diseases, 11% have symptoms of osteoarthritis [2].

Osteoarthritis is usually a slowly progressive disease that affects the entire joint, including articular cartilage, subchondral bone, ligaments, synovium, and even adjacent muscles [9,10,11,12]. The etiology of degenerative changes in the temporomandibular joints is complex. According to Arnett [13,14], degenerative changes occur as a result of disturbed remodeling of the temporomandibular joint. Remodeling is the basic biological response to loading the temporomandibular joint, ensuring mutual balance between the joint, function, and occlusion. Incorrect remodeling can occur as a result of a reduction in the adaptability of the temporomandibular joints, as well as due to excessive or prolonged overload of the temporomandibular joints [2,13,14].

In the pathogenesis of osteoarthritis, the mechanical, inflammatory, and metabolic factors are considered to be responsible for the damage of synovial joints [11,12]. The initiation and progression of the temporomandibular joint osteoarthritis may be influenced by injuries (as a result of injuries, the mechanical properties of the articular disc change, cartilage degradation, and the production of inflammatory and pain mediators), parafunctions (as a result of which dislocation of the articular disc may occur, as well as degenerative changes within the condyle and articular eminence), unstable occlusion, functional overload, and increased friction within the temporomandibular joint itself [2]. Genetic components are also discussed to be involved in the pathogenesis of TMJ osteoarthritis, i.e.: the expression of transforming growth factor-ß1 [15]. Furthermore, mRNA expression of the biomarkers: EGR1 (early growth response 1), EPHX1 (epoxide hydrolase 1), and IL10 (interleukin 10) coming from peripheral blood leukocytes are involved in the joint tissue damage and repair [16].

The most common symptom of degenerative changes in the temporomandibular joints is joint pain [2,17,18,19]. It comes from the soft tissues surrounding the joint and from the masticatory muscles. Muscle spasm of the masticatory organ is a physiological defensive reflex that protects the damaged joint from its further destruction. One of the clinical manifestations of the temporomandibular joints’ osteoarthritis is joint pain both when opening the mouth and during lateral movements, as well as crepitus [20]. Other symptoms associated with degenerative changes in the temporomandibular joints include the impairment of normal joint function, ankylosis, joint instability, as well as facial deformity, caused by the decrease of posterior facial height, which occurs as a result of the condyle osteolysis [2]. However, the temporomandibular joint osteoarthritis may also be completely asymptomatic [21,22].

Temporomandibular joint osteoarthritis is diagnosed on the basis of radiographic examination. Among all of the imaging methods, cone beam computed tomography (CBCT) has high diagnostic value in the assessment of the bony structures of the temporomandibular joints. CBCT is an alternative to conventional computed tomography (CT) but with a lower radiation dose, lower image contrast, and at the same time with higher radiographic noise. CBCT of the temporomandibular joint, among many advantages, improves the qualitative analysis of the condylar surface, allows detecting the shape of the mandibular condyle, and improves the accuracy of linear measurements of mandibular condyle [23].

The study aimed to compare the morphology of the temporomandibular joints, including the mandibular condyle, glenoid fossa, and articular eminence, as well as the position of the condyle in glenoid fossa in cone beam computed tomography (CBCT) images regarding the number of the osteoarthritic changes diagnosed in the area of the condyle. The primary outcome was the assessment of the impact of the osteoarthritic changes on the temporomandibular joint’s morphology.

## 2. Materials and Methods

### 2.1. Participants

One hundred and five participants with no complaint for any disease (79 women and 26 men; mean age: 24.93 ± 7.74 years) who had come to the specialist orthodontic practice for consultation engaged in the study. None of the patients had ever undergone orthodontic treatment. The total number of examined temporomandibular joints was 210. Inclusive criteria were as follows: age between 16 and 60 years old, willingness to participate in the study, and people who were generally healthy and had never been treated orthodontically. Exclusion criteria were as follows: age below 16 and above 60 years old, anterior disc displacement without reduction, temporomandibular joint ankylosis, pregnancy, systemic rheumatic diseases, oncological diseases, people who had undergone radiotherapy (especially in the area of head and neck), reported trauma from the patients in the field of head and neck, people who had been treated orthodontically at least once in the past, also these who did not agree to take part in the study. All patients received and signed informed consent. The study was approved by the Independent Bioethics Committee for Scientific Research and was conducted with the ethical principles of the World Medical Association Declaration of Helsinki.

### 2.2. Protocol

Each patient underwent standard pre-orthodontic treatment examination, which included the following: general health questionnaire, anamnesis, extraoral and intraoral examination, extraoral and intraoral photos, plaster casts, radiographs, orthopantomogram, and latera cephalometric radiography. Since many patients who are clinically asymptomatic may present radiographic symptoms of the temporomandibular joint osteoarthritis, the standard orthodontic examination was extended by taking additional cone beam computed tomography (CBCT) images of the temporomandibular joints.

**Figure 1:**
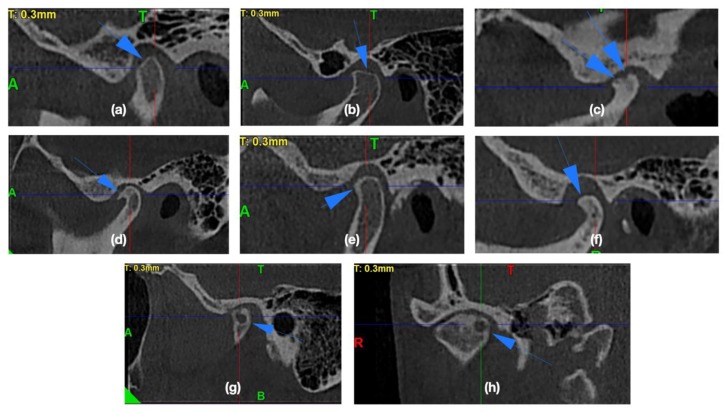
The exemplary osteoarthritic changes found in the temporomandibular joints (TMJ) cone beam computed tomography (CBCT) scans: (**a,b**) Articular surface flattening; (**c**) Erosion; (**d**) Osteophyte; (**e**) Subcortical sclerosis; (**f**) Generalized sclerosis; (**g,h**) Subcortical cyst.

In the CBCT images, the presence of the osteoarthritic changes was assessed. The radiographic symptoms of degenerative joint disease are [20,24,25,26,27,28,29] as follows: flattening of the convex condylar head; erosion (the area of reduced density within the cortex and subcortical bone); osteophytes (bone outgrowths on the surface of the condyles); sclerosis (increased density of the cortical plate or bone tissue under the cortical plate); pseudocysts (osteolytic, well delimited, localized in the subcortical area, the cortical layer does not become destroyed in its course). Figure 1 presents the exemplary osteoarthritic changes found in the TMJ CBCT scans.

The temporomandibular joints were allocated into one of three groups regarding the number of diagnosed osteoarthritic changes: Group 1 – control group (with no or 1 type of osteoarthritic changes), Group 2 (2 different types of osteoarthritic changes), Group 3 (3 or more different types of osteoarthritic changes).

### 2.3. Imaging Procedures

Cone beam computed tomography (CBCT) imaging was conducted on a MyRay Hyperion X9 3D. The parameters of exposition were 90 kV, 18 mAs, and an exposition time of 3.6 s. The established field of view (FOV) was 8 cm × 5 cm, and the thickness of slices was 0.3 mm. The radiation dose was limited by the reduction of the exposition time, reduction of the field of view, and increase of the thickness of slices to the values that enable achieving diagnostic images according to the concept of “as low as diagnostically acceptable” (ALADA). While the CBCT scans were being taken, patients were standing straight, holding their heads upright, looking directly into their eyes’ reflection in the mirror hanging in front of them. Patients held their teeth in the position of maximum intercuspation.

### 2.4. Measurements

**Figure 2:**
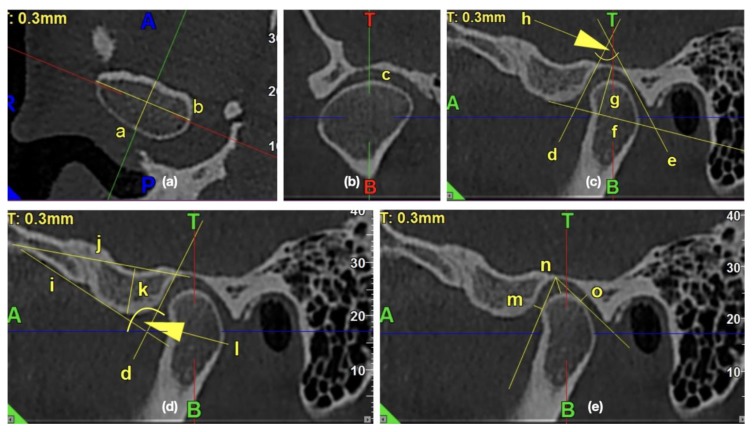
The exemplary lines, points, and angles in the temporomandibular joints (TMJ) cone beam computed tomography (CBCT) scans used for measurements. (**a**) Morphology of mandibular condyle in the axial view: /a/condylar A-P dimension, /b/condylar width; (**b**) Shape of condyle head in the coronal view: /c/condyle’s superior surface view assessment; (**c**) Morphology of glenoid fossa in the sagittal view: /d/PE line, /e/AT line, /f/glenoid fossa basal line, /g/glenoid fossa depth, /h/glenoid fossa divergence angle; (**d**) Morphology of articular eminence in the sagittal view: /i/AE line, /j/eminence basal line, /k/articular eminence height, /l/articular eminence divergence angle; (**e**) Assessment of anterior, posterior, and superior joint spaces in the sagittal view: /m/anterior joint space, /n/superior joint space, /o/posterior joint space.

All the measurements were performed with the use of iRYS Softwarwe version 6.2. The primary outcome was the assessment of the impact of the osteoarthritic changes on the temporomandibular joint’s morphology. The 0.3-mm thickness axial slice of the condyle, in which the condyle had the maximum mediolateral dimension, was selected for further measurements. The sagittal axis was established as a line, which was perpendicular to and, at the same time, crossing the middle of the line connecting the mesial and distal end of the condyle. The obtained sagittal and coronal images were further examined and measured. Figure 2 presents the exemplary lines, points, and angles in the TMJ CBCT scans used for measurements.

#### 2.4.1. Morphology of the Mandibular Condyle

According to Yale’s classification based on the condyle’s superior surface view, each condyle was classified as one of four types: convex, flattened, angled, and rounded. The shape of the condyle head was assessed in the obtained coronal image.

Condylar width was a maximum mediolateral width measured in the axial image.

The condylar A-P dimension was measured from the most anterior to the most posterior point on the condylar head as a perpendicular distance to maximum mediolateral width, crossing it in the middle.

#### 2.4.2. Morphology of the Glenoid Fossa (Mandibular Fossa, Articular Fossa)

The shape of glenoid fossa was assessed in the obtained sagittal slices. The classification of shapes of the fossae included oval, triangular, angled, trapezoidal, and other types.

Glenoid fossa depth was measured as a perpendicular distance from the highest point of the glenoid fossa to the fossa basal line in the obtained sagittal image.

The fossa basal line was traced from the lowest point of the articular eminence to the lowest point of external auditory meatus in the obtained sagittal image.

The glenoid fossa length was measured from the lowest point of the articular eminence to the anterior part of the tympanic part of the temporal bone along the basal line, connecting the lowest point of the articular eminence with the lowest point of the external auditory meatus.

Glenoid fossa divergence angle was the angle measured between two lines: PE (posterior eminence line) and AT (anterior tympanic line) in the obtained sagittal images.

PE line was traced as the best fitting line, which was tangent to the posterior wall of the articular eminence.

AT line was traced as the best fitting line, which was tangent to the anterior wall of the tympanic part of the temporal bone.

#### 2.4.3. Morphology of the Articular Eminence

Articular eminence height was measured as a perpendicular distance from the lowest point on an articular eminence to the eminence basal line, which was measured in the obtained sagittal image.

The eminence basal line was traced from the highest point of articular fossa and tangent to the base of the articular eminence.

The articular eminence divergence angle was the angle measured between two lines: AE (anterior eminence line) and PE (posterior eminence line) in the obtained sagittal images.

The AE line was traced as the best fitting line, which was tangent to the anterior wall of the articular eminence.

The PE line was traced as the best fitting line, which was tangent to the posterior wall of the articular eminence.

#### 2.4.4. Assessment of the Anterior, Posterior, and Superior Joint Spaces

Joint spaces were measured in the obtained sagittal slices. From the highest point of glenoid fossa, two lines were traced, one of which approached the most posterior point of the condyle (CP-line), whereas the latter reached the most anterior point of the condyle (CA-line).

The anterior joint space was perpendicular to the CA-line distance measured from the most anterior point of the condyle to the glenoid fossa.

The posterior joint space was perpendicular to the CP-line distance measured from the most posterior point of the condyle to the glenoid fossa.

The superior joint space was the distance measured from the most superior point of glenoid fossa to the most superior point on the condylar head.

#### 2.4.5. Assessment of the Sagittal Position of the Condyle

The sagittal view of the condyle was assessed according to Pullinger and Hollender’s formula [30]:

(1) $$condylar\;radio\;=\;\frac{P-A}{P+A}\times 100\%$$

where:

P—posterior joint space;
A—anterior joint space.
The concentric position of the condyle was diagnosed if the condylar ratio was ±12%.
The posterior position of the condyle was diagnosed if the condylar ratio was <−12%.
Anterior position of the condyle was diagnosed if the condylar ratio was >12%.

#### 2.4.6. Statistical Analysis

Statistical analysis was performed with StatSoft.Inc. (2014) STATISTICA (data analysis software system) version 12.0. www.statsoft.com. The mean value, standard deviation, median, minimum value, maximum value, and 95%CI (confidence interval) were measured. The Shapiro–Wilk test was performed to check the normality of the distribution. To check the equality of group variances, the Brown–Fosythe test was used. The T-Student test, Welch test, or U Mann–Whitney tests were chosen according to the indications to check the significance of the differences between two different groups. When more than two groups were to be compared, either test F (ANOVA) or the Kruskal–Wallis test was used. When the differences among the groups were statistically significant, the post hoc tests were performed respectively: Tukey test for ANOVA and Dunn’s test for the Kruskal–Wallis test. To check whether two qualitative variables are independent, the Chi-square test of independence was used (with Yates’s correction, Cochran’s test, and Fisher’s exact test). Pearson correlation coefficient and/or Spearman’s rank correlation coefficient were measured to assess the correlation between two variables. The statistical significance level was *p* = 0.05 for all the measurements included.

## 3. Results

### 3.1. General Characteristics of the Examined Patients

Two hundred and ten temporomandibular joints (TMJs) from the 105 patients (79 women and 26 men; average age ± SD: 24.93 ± 7.74; range of age: 16–47 years old) were analyzed. There was significant correlation between right and left temporomandibular joints regarding the number of osteoarthritic changes R = 0.44, *p* = 0.0001. There were 70 TMJs with no or one type of osteoarthritic change (Group 1), 79 TMJs with 2 different types of osteoarthritic changes (Group 2), and 61 TMJs with 3 or more different types of osteoarthritic changes (Group 3). Table 1 presents the comparison of age among the examined groups.

Among all of the TMJs, the articular surface flattening was the most common type of the osteoarthritic changes (it occurred in 90% of all TMJs). The frequency of other osteoarthritic changes was as presented below: surface erosion (41.9%), subcortical sclerosis (33.3%), osteophyte (27.1%), subcortical cyst (11.0%), and the least frequent was the generalized sclerosis (1% of all TMJs). Fourteen temporomandibular joints were interpreted as normal with no symptoms of osteoarthritis. Table 2 presents general characteristics of the examined TMJs regarding the type and the number of diagnosed osteoarthritic changes.

There were 70 temporomandibular joints with up to one type of osteoarthritic change (Group 1), 79 TMJs with 2 different types of osteoarthritic changes (Group 2), and 61 TMJs with 3 or more different types of osteoarthritic changes (Group 3). There were significant differences regarding the distributions of osteoarthritic changes among the examined groups. In Group 1, 74.3% of examined TMJs presented articular surface flattening. None of the TMJs from Group 1 was diagnosed with osteophyte, subcortical cyst, or generalized sclerosis. In Group 2, articular surface flattening occurred in 98.7% of the examined TMJs. The number of cases in Group 2 with surface erosion and subcortical sclerosis increased. There also appeared one TMJ with subcortical cyst. In Group 3, the frequency of articular surface flattening among the examined TMJs was similar to that in Group 2: articular surface flattening was present in nearly all examined TMJs (96.7%). There was also the highest observed increase in the frequency of osteophytes (from 16.5% in Group 2 to 72.1% in Group 3) and subcortical cysts (from 1.3% in Group 2 to 36.1% in Group 3) compared to Group 2. Furthermore, there were two TMJs diagnosed with generalized sclerosis in Group 3. Table 3 presents the distributions of the osteoarthritic changes among the examined groups.

Table 4 presents comparable characteristics of the condyle’s head, glenoid fossa, and articular eminence, as well as condylar head position in glenoid fossa regarding the number of the osteoarthritic changes diagnosed in the area of the condyle.

### 3.2. Condyle Head

There were no statistically significant differences regarding the distribution of the condyle head’s shapes among the examined groups (*p* = 0.2824).

The average condylar width in the control group (Group 1) was 19.0 (2.3) mm (range: 13.9–23.7 mm), in the group with two different types of osteoarthritic changes (Group 2), it was 18.9 (2.1) mm (range: 13.4–23.9 mm), whereas in the group with three or more different types of osteoarthritic changes (Group 3), it was 18.7 (2.1) mm (range: 11.7–22.1 mm). There were no statistically significant differences regarding the condylar width between the examined groups (*p* = 0.8416).

The average condylar A-P dimension in the control group (Group 1) was 7.3 (1.3) mm (range: 4.4–11.7 mm), in the group with two different types of osteoarthritic changes (Group 2), it was 6.6 (1.2) mm (range: 3.2–9.7 mm), whereas in the group with three or more different types of osteoarthritic changes (Group 3), it was 5.8 (1.3) mm (range: 2.3–8.7 mm). The condylar A-P dimension differed significantly among the groups (*p* = 0.0001). The condylar A-P dimension was significantly higher in Group 1 comparing to both Group 2 (*p* = 0.0065) and Group 3 (*p* = 0.0001), and it was also significantly higher in Group 2 compared to Group 3 (*p* = 0.0025).

### 3.3. Glenoid Fossa

There were no statistically significant differences regarding the distribution of the glenoid fossa’s shapes among the examined groups (*p* = 0.1678).

The average depth of the glenoid fossa in the control group (Group 1) was 9.8 (1.3) mm (range: 6.3–12.1 mm), and in the group with two different types of osteoarthritic changes (Group 2), it was 9.6 (1.5) mm (range: 6.6–12.9 mm), whereas in the group with three or more different types of osteoarthritic changes (Group 3), it was 9.8 (1.3) mm (range: 6.9–12.7 mm). There were no statistically significant differences regarding the depth of the glenoid fossa among the examined groups (*p* = 0.5523).

The average length of glenoid fossa in the control group (Group 1) was 20.9 (2.2) mm (range: 16.5–27.0 mm), and in the group with two different types of osteoarthritic changes (Group 2), it was 20.2 (2.3) mm (range: 15.6–28.3 mm), whereas in the group with three or more different types of osteoarthritic changes (Group 3), it was 20.0 (2.0) mm (range: 16.1–25.3 mm). There were no statistically significant differences regarding the length of the glenoid fossa among the examined groups (*p* = 0.6834).

The average divergence angle of glenoid fossa in the control group (Group 1) was 57.5° (15.1°) (range: 29.2–100.5°), in the group with two different types of osteoarthritic changes (Group 2), it was 55.7° (14.7°) (range: 26.0–94.2°), whereas in the group with three or more different types of osteoarthritic changes (Group 3), it was 55.6° (12.8°) (range: 34.7–97.8°). There were no statistically significant differences regarding the divergence angle of the glenoid fossa among the examined groups (*p* = 0.6037).

### 3.4. Articular Eminence

The average height of the articular eminence in the control group (Group 1) was 8.1 (2.1) mm (range: 3.6–13.6 mm), while in the group with two different types of osteoarthritic changes (Group 2), it was 8.2 (2.2) mm (range: 3.0–13.7 mm), and in the group with three or more different types of osteoarthritic changes (Group 3), it was 7.9 (1.9) mm (range: 3.4–12.0 mm). There were no statistically significant differences regarding the height of articular eminence among the examined groups (*p* = 0.8336).

The average divergence angle of the articular eminence in the control group (Group 1) was 82.1° (15.4°) (range: 49.7–127.6°), in the group with two different types of osteoarthritic changes (Group 2), it was 81.1º (16.3°) (range: 43.7–118.4°), whereas in the group with three or more different types of osteoarthritic changes (Group 3), it was 84.9° (12.6°) (range: 57.6–112.1°). There were no statistically significant differences regarding the divergence angle of articular eminence between the among groups (*p* = 0.1548).

### 3.5. Assessment of the Sagittal Position of the Condyle

There were no significant differences regarding the condylar ratio (according to the Pullinger and Hollender’s formula) among the examined groups (*p* = 0.2266). Despite the fact that there were also no significant differences regarding the distribution of the sagittal position of the condyle in the glenoid fossa, there was an increasing tendency in the frequency of posterior position of the condyle with the greater amount of diagnosed osteoarthritic changes.

## 4. Discussion

Diagnostic Criteria for Temporomandibular Disorders (DC/TMD) for Clinical and Research Applications indicate that imaging is the reference standard for the diagnosis of degenerative joint disease (DJD). The diagnosis based only on clinical examination without imaging has a sensitivity of 0.55 and a specificity of 0.61 [31]. Cone beam computed tomography (CBCT) is dedicated for the diagnosis of the temporomandibular joint bony structures, including assessment of the shape of the joint surfaces, the head of the condyle, and the width of the articular space [1,21,23,25,27]. There are three major radiographic symptoms of DJD: subcortical pseudocyst, osteophyte, and surface erosion. Articular surface flattening and subcortical sclerosis may be related to the osseous remodeling, but they may also lead to DJD development over time [32].

This is the first study that not only focuses on the frequency of the temporomandibular joints’ osteoarthritic changes, but also quantitatively analyzes the morphology of the temporomandibular joints regarding the number of diagnosed osteoarthritic changes in the area of the condyle.

In the presented study, there were no significant differences regarding the age among the examined groups. Similar results were obtained by Walewski et al. [33] and Al-Ekrish et al. [34]. Nonetheless, Alexiou et al. [29] confirmed that osteoarthritic changes were related to age. The disagreement between our results may be the consequence of differences regarding the size and age between groups included into the study. Not only was the group examined by Alexiou smaller (71 people), but also the average age was much higher (48.17 years old).

Osteoarthritis is considered to be the most common joint disease [12]. From the examined group of 210 temporomandibular joints (TMJs), only 14 had no symptoms of osteoarthritis. In the literature, the authors diagnosed osteoarthritic changes with different frequency. Shahidi et al. [35] found the osteoarthritic changes in 90% TMJs of patients with symptoms of the temporomandibular joint disorders (TMD) and 86.7% TMJs of asymptomatic subjects, which is close to our results. Ottersen et al. [36] confirmed osteoarthritis in nearly half of the examined TMJs, whereas Bakke et al. [37] observed degenerative changes in 39.3% of the examined TMJs. Both Ottersen et al. [36] and Bakke et al. [37] diagnosed TMJs osteoarthritis only when the condylar deformation with the presence of either subcortical cyst, surface erosion, osteophyte, or generalized sclerosis was observed. Temporomandibular joints with surface flattening, subcortical sclerosis, or even subcortical cyst, surface erosion, osteophyte or generalized sclerosis, but with no condylar deformation, were diagnosed as indeterminate for osteoarthritis. According to Ottersen et al. [36], slight articular surface flattening and subcortical sclerosis should be interpreted as a normal variant. Nonetheless, Ahmad and Schiffmann [31] indicated that both the articular surface flattening and subcortical sclerosis may transform to osteoarthritis. Taking all of these into consideration, the initial osteoarthritic changes should be considered as risk factor for osteoarthritis and would require long-term thorough observations.

According to this research, the most common osteoarthritic changes were articular surface flattening (90.0% of examined joints) and surface erosion (41.9% of examined joints). Although the articular surface flattening was also confirmed as the most common osteoarthritic change by other authors [35,36,37], it was most often considered as an indeterminate change of osteoarthritis. Ottersen et al. [36] found that articular surface flattening (79%), osteophyte (72%), and subcortical sclerosis (70%) were three most common osteoarthritic changes in condyle. The authors also assessed the combination of osteoarthritic changes, among which the most common was articular surface flattening and osteophyte formation together with flattening of the fossa/eminence. According to Bakke et al. [37], articular surface flattening was the most commonly diagnosed osseous change. Similar observations were presented in Shahidi et al. [35], who also found articular surface flattening as the most common osteoarthritic change.

The majority of published research studies focused only on the recognition of the TMJ osteoarthritic changes in the CBCT images. There are only a few studies in the literature that attempted to assess the mandibular and temporomandibular-joint morphology regarding the presence of osteoarthritic changes.

In the presented study, we have observed significant decrease in the condylar anteroposterior dimension with the increase in the number of osteoarthritic changes. The relationship between the condylar anteroposterior dimension and the number of diagnosed osteoarthritic changes has not been discussed in the literature yet. Cho et al. [38] compared the mandibular morphology between 39 patients with TMD and 44 asymptomatic people. The authors diagnosed osteoarthritis on the basis of CT images but performed all the measurements regarding the morphology of the mandible on panoramic radiographs. They found that in the “osteoarthritic group”, the condylar head, condylar height, and ramal height were significantly shorter, the gonial angles were significantly larger, and the condylar head presented a more distal inclination compared to the control group. The authors suggested that distally inclined condyles were the result of osteoarthritic deformity of the condyle in the anterosuperior area. These observations follow our results. It is probable that the bigger the number of osteoarthritic changes, the more severe the condylar destruction in anterosuperior area. Consequently, the anteroposterior dimension becomes diminished and the condylar head becomes inclined more distally.

We found no significant differences regarding the height of the articular eminence among the examined groups. Similar results were obtained by Ilguy et al. [39]. The authors assessed the articular eminence inclination and height regarding the presence of the osteoarthritic changes. The articular eminence inclination was measured by two different methods: first, the best-fit line method, which measured the angle between Ebf (best-fit line) and Frankfort horizontal, and second, the top-roof line method, which measured the angle between Etr (top-roof line) and Frankfort horizontal. The authors measured the eminence height as a perpendicular distance between the highest point of glenoid fossa and the lowest point of the articular eminence. Despite the fact that the methodology used in ours studies differed, the authors also found no significant differences between the articular eminence height and the presence of the osteoarthritic changes. There were also no significant differences between the articular eminence inclination (both methods) and the presence of the osteoarthritic changes.

In addition to the abovementioned results, Ilguy et al. [39] also measured the thickness of the roof of the glenoid fossa (RGF) and found significant correlation between the thickness of RGF and sagittal condyle morphology with the thickest RGF in osteoarthritic patients with osteophytes. However, at the same time, osteoarthritic patients with the articular surface flattening had slightly reduced average RGF comparing to the patients without osteoarthritis. This could have happened if the patients with the articular surface flattening, in fact, had had no osteoarthritis, but presented symptoms of typical bone remodeling. The thickness of RGF was also measured by Ejima et al. [40]. They found that groups with osteoarthritis had higher RGF thickness than the temporomandibular joints without osteoarthritis. According to the studies by Honda et al. [41], an increase in bone thickness in the glenoid fossa may be caused by mechanical stimulation because of an incomplete shock absorption function resulting from the perforation of the disc or altered retrodiscal connective tissue. In our research, there were no significant differences between the presence of the osteoarthritic changes and the height, length, or divergence angle of the glenoid fossa. We did not measure the thickness of the roof of the glenoid fossa. Nonetheless, if the thickness of RGF had increased in the osteoarthritic cases, it is probable that the height of the glenoid fossa would have changed, but this was not observed.

This study has some potential limitations. First of all, we diagnosed as the osteoarthritic changes all of the below mentioned: flattening of the convex condylar head, erosion, osteophytes, sclerosis, and pseudocysts. Some of the authors do not consider the articular surface flattening as the osteoarthritic change unless the condylar deformation is present. Second, the age of the participants is limited to range: 16–47 years old. Although we analyzed 210 TMJs, it would be valuable to examine a larger group, including elderly individuals. Third, this particular study was based on the CBCT images with the moderate FOV (8 cm × 5 cm). This FOV does not allow to take the measurements that refer to the Frankfort horizontal plane, because the anterior part of the orbital floor is not covered by the slices. A bigger FOV would allow increasing the number of measurements to examine the temporomandibular joint’s morphology more thoroughly.

## 5. Conclusions

The temporomandibular joints’ osteoarthritic changes occur very often even among asymptomatic patients. The most common osteoarthritic change was articular surface flattening. The increased number of osteoarthritic changes seems to have an impact on the condylar anteroposterior dimension. The bigger the number of osteoarthritic changes diagnosed in one joint, the smaller the condylar anteroposterior dimension that was observed.

## Figures and Tables

**Table 1:** Comparison of age among the examined groups.

Comparable Characteristic	Group 1	Group 2	Group 3	*p*-Value
**AGE**				0.296046 ^1^
av. (SD)	23.5 (6.3)	25.2 (8.1)	26.4 (8.9)	
range	16.1–39.7	16.3–45.6	16.0–47.0	
median	21.4	23.3	25.1	
95%CI	[21.4;25.6]	[22.4;27.9]	[23.1;29.8]	

^1^ ANOVA (test F).

**Table 2:** General characteristics of the examined temporomandibular joints regarding the type and the number of diagnosed osteoarthritic changes.

Osteoarthritic Change	Number of TMJs (%)
Subcortical sclerosis	70 (33.3%)
Osteophyte	57 (27.1%)
Subcortical cyst	23 (11.0%)
Surface erosion	88 (41.9%)
Articular surface flattening	189 (90.0%)
Generalized sclerosis	2 (1.0%)
None	14 (6.7%)

**Table 3:** Distributions of the osteoarthritic changes among the examined groups.

Osteoarthritic Change	Group 1 (N = 70)	Group 2 (N = 79)	Group 3 (N = 61)	*p*-Value
Subcortical sclerosis	2 (2.9%)	28 (35.4%)	40 (65.6%)	0.0001 ^1^
Osteophyte	0 (0.0%)	13 (16.5%)	44 (72.1%)	0.0001 ^1^
Subcortical cyst	0 (0.0%)	1 (1.3%)	22 (36.1%)	0.0001 ^1^
Surface erosion	2 (2.9%)	38 (48.1%)	48 (78.7%)	0.0001 ^1^
Articular surface flattening	52 (74.3%)	78 (98.7%)	59 (96.7%)	0.0001 ^1^
Generalized sclerosis	0 (0.0%)	0 (0.0%)	2 (3.3%)	0.0849 ^1^

^1^ Chi square.

**Table 4:** Comparable characteristics of the condyle’s head, glenoid fossa, and articular eminence, as well as condylar head position in glenoid fossa regarding the number of the osteoarthritic changes diagnosed in the area of the condyle.

Comparable Characteristics	Group 1	Group 2	Group 3	*p*-Value
	(N = 70)	(N = 79)	(N = 61)	
**CONDYLE**				
**Shape of condyle head**				0.2824 ^3^
flattened	11 (15.7%)	24 (30.4%)	14 (23.0%)	
convex	30 (42.9%)	25 (31.6%)	25 (41.0%)	
angled	9 (12.9%)	14 (17.7%)	11 (18.0%)	
round	20 (28.6%)	16 (20.3%)	11 (18.0%)	
**Condylar width [mm]**				0.8416 ^1^
av. (SD)	19.0 (2.3)	18.9 (2.1)	18.7 (2.1)	
range	13.9–23.7	13.4–23.9	11.7–22.1	
median	19.2	19.0	18.8	
95%CI	[18.5; 19.6]	[18.4; 19.4]	[18.2; 19.2]	
**Condylar A-P dimension [mm]**				0.0001 ^2^
av. (SD)	7.3 (1.3)	6.6 (1.2)	5.8 (1.3)	
range	4.4–11.7	3.2–9.7	2.3–8.7	^a^0.0065
median	7.2 ^a,b^	6.6 ^a,c^	6.0 ^b,c^	^b^ 0.0001
95%CI	[7.0; 7.6]	[6.3; 6.9]	[5.5; 6.1]	^c^ 0.0025
**GLENOID FOSSA**				
**Shape**				0.1678 ^3^
oval	36 (51.4%)	50 (63.3%)	44 (72.1%)	
trapezoidal	19 (27.1%)	14 (17.7%)	13 (21.3%)	
triangular	10 (14.3%)	11 (13.9%)	3 (4.9%)	
angled	5 (7.1%)	4 (5.1%)	1 (1.6%)	
**Depth [mm]**				0.5523 ^1^
av. (SD)	9.8 (1.3)	9.6 (1.5)	9.8 (1.3)	
range	6.3–12.1	6.6–12.9	6.9–12.7	
median	10.0	9.5	9.9	
95%CI	[9.5;10.1]	[9.3;9.9]	[9.5;10.1]	
**Length [mm]**				0.6834 ^2^
av. (SD)	20.9 (2.2)	20.2 (2.3)	20.0 (2.0)	
range	16.5–27.0	15.6–28.3	16.1–25.3	
median	20.5	20.1	19.9	
95%CI	[20.4;21.4]	[19.7;20.7]	[19.5;20.5]	
**Divergence angle [°]**				0.6037 ^2^
av. (SD)	57.5 (15.1)	55.7 (14.7)	55.6 (12.8)	
range	29.2–100.5	26.0–94.2	34.7–97.8	
median	58.8	55.1	55.0	
95%CI	[53.9;61.1]	[52.4;58.9]	[52.3;58.8]	
**ARTICULAR EMINENCE**				
**Height [mm]**				0.8336 ^1^
av. (SD)	8.1 (2.1)	8.2 (2.2)	7.9 (1.9)	
range	3.6–13.6	3.0–13.7	3.4–12.0	
median	8.2	8.4	8.1	
95%CI	[7.7;8.6]	[7.7;8.6]	[7.4;8.4]	
**Divergence angle [°]**				0.1548 ^1^
av. (SD)	82.1 (15.4)	81.1 (16.3)	84.9 (12.6)	
range	49.7–127.6	43.7–118.4	57.6–112.1	
median	79.9	79.8	86.1	
95%CI	[78.5;85.8]	[77.4;84.7]	[81.6;88.1]	
**CONDYLAR HEAD POSITION**				
**Superior [mm]**				0.3101 ^2^
av. (SD)	3.2 (0.9)	3.3 (0.9)	3.1 (1.0)	
range	1.2–5.8	1.5–6.1	1.2–6.5	
median	3.2	3.3	3.1	
95%CI	[3.0; 3.5]	[3.1; 3.5]	[2.8; 3.4]	
**(P − A)/(P + A) [%]**				0.2266 ^2^
av. (SD)	−2.1 (20.6)	−8.7 (23.4)	−6.2 (24.3)	
range	−54.1–47.6	−69.4–56.0	−55.8–60.9	
median	0.0	−4.3	−6.7	
95%CI	[−7.0; 2.8]	[−13.9; −3.5]	[−12.4; 0.1]	
**Position**				0.5974 ^3^
anterior	15 (21.4%)	14 (17.7%)	13 (21.3%)	
posterior	23 (32.9%)	29 (36.7%)	27 (44.3%)	
concentric	32 (45.7%)	36 (45.6%)	21 (34.4%)	

^1^ ANOVA (test F), ^2^ Kruskal–Wallis, ^a,b,c^ post-hoc Dunn’s test,^3^ Chi square.
